# Systemic inflammatory indices and the FT4/TSH ratio in Hashimoto’s thyroiditis: predictive and prognostic implications

**DOI:** 10.1007/s11845-026-04409-9

**Published:** 2026-04-22

**Authors:** Ecem Demir, Şeyma Taştemur

**Affiliations:** 1 Department of Statistics and Computer Sciences, Faculty of Science, Sivas Cumhuriyet University, Sivas, Türkiye; 2 Department of Internal Medicine, Faculty of Medicine, Sivas Cumhuriyet University, Sivas, Türkiye

**Keywords:** Hashimoto’s thyroiditis, FT4/TSH ratio, Lymphocyte-to-monocyte ratio, Autoimmune thyroid disease, Inflammatory indices

## Abstract

**Objective:**

To evaluate thyroid-related parameters and inflammation-derived hematological indices in patients with Hashimoto’s thyroiditis compared with healthy controls, and to assess their diagnostic performance across different thyroid functional states.

**Methods:**

This retrospective case–control study included 92 patients with Hashimoto’s thyroiditis and 92 healthy controls. Thyroid function tests, thyroid autoantibodies, inflammatory markers, and complete blood count–derived indices were analyzed. Patients were further classified as overt hypothyroid, subclinical hypothyroid, or euthyroid. Receiver operating characteristic (ROC) analysis and multivariable logistic regression were performed to assess diagnostic performance and identify independent predictors.

**Results:**

Patients showed significantly altered thyroid hormone profiles and markedly higher thyroid autoantibody levels than controls (all *p* < 0.001). Among hematological parameters, erythrocyte sedimentation rate, monocyte count, hemoglobin level, and the lymphocyte-to-monocyte ratio (LMR) differed significantly, whereas composite indices such as the neutrophil-to-lymphocyte ratio, platelet-to-lymphocyte ratio, and systemic immune-inflammation index did not. Thyroid hormone–related parameters, particularly the FT4/TSH ratio, clearly distinguished clinical subgroups, while autoantibody and inflammatory indices were comparable across functional states. In multivariable analysis, the FT4/TSH ratio and LMR emerged as independent predictors of patient status.

**Conclusion:**

Routine laboratory ratios, especially the FT4/TSH ratio and LMR, provide complementary diagnostic information alongside established thyroid markers and may enhance clinical assessment of Hashimoto’s thyroiditis.

## Introduction

Hashimoto’s thyroiditis is a chronic autoimmune disorder and the leading cause of hypothyroidism in iodine-sufficient populations. It is characterized by immune-mediated destruction of thyroid tissue and occurs predominantly in women. The clinical presentation is heterogeneous, spanning from preserved thyroid function to subclinical and overt hypothyroidism [1, 2]. While alterations in thyroid hormone levels form the basis of clinical diagnosis, growing evidence suggests that Hashimoto’s thyroiditis represents a systemic immune-mediated condition rather than an isolated endocrine disorder [3].

The immunopathogenesis of Hashimoto’s thyroiditis involves loss of self-tolerance and activation of autoreactive lymphocytes, resulting in the production of thyroid-specific autoantibodies. Anti-thyroid peroxidase (Anti-TPO) and anti-thyroglobulin (anti-TG) antibodies are widely used diagnostic markers and are detected in most patients [4]. Nevertheless, antibody titers may vary considerably, and a proportion of patients remain seronegative, indicating that autoantibody status alone may not fully reflect disease activity or progression [5].

In parallel, interest has increased in systemic inflammatory markers derived from routine hematological parameters. Indices such as the neutrophil-to-lymphocyte ratio (NLR), platelet-to-lymphocyte ratio (PLR), lymphocyte-to-monocyte ratio (LMR), and the systemic immune-inflammation index (SII) integrate information from different immune cell populations and have been proposed as indicators of chronic low-grade inflammation [6]. These markers have shown clinical relevance in several autoimmune and inflammatory diseases and, in some settings, have demonstrated greater sensitivity than traditional acute-phase reactants [7].

Despite growing interest, the clinical relevance of inflammation-derived hematological indices in Hashimoto’s thyroiditis, particularly across different thyroid functional states, remains unclear; therefore, this study aimed to evaluate these indices alongside thyroid-related parameters in patients and healthy controls.

## Materials and methods

This retrospective, data-based observational case–control study compared patients diagnosed with Hashimoto’s thyroiditis with healthy controls on hematological indices reflecting inflammation and thyroid-related parameters. Patients were classified as having Hashimoto’s thyroiditis based on a documented clinical diagnosis in the medical record made by an endocrinology/internal medicine specialist plus objective evidence of autoimmune thyroiditis. Objective evidence required at least one of the following: (i) positive thyroid autoantibodies (anti-TPO and/or anti-TG above the institutional upper reference limit; anti-TPO > 34 IU/mL and/or anti-TG > 115 IU/mL), and/or (ii) characteristic thyroid ultrasonography findings consistent with chronic autoimmune thyroiditis (e.g., diffuse hypoechogenicity and heterogeneous parenchymal echotexture) reported by radiology/endocrinology.

Patients were excluded if records suggested alternative etiologies of thyroid dysfunction, including Graves’ disease/toxic nodular disease, subacute thyroiditis, postpartum thyroiditis, prior thyroid surgery or radioiodine therapy, current amiodarone or interferon use, or insufficient data to confirm the above criteria, acute infection or active inflammatory conditions, malignancy, systemic autoimmune diseases other than Hashimoto’s thyroiditis, hematological disorders, pregnancy, severe renal or hepatic failure, and the use of medications such as immunosuppressive agents or corticosteroids that could significantly affect complete blood count parameters or inflammatory markers. Individuals aged 18–65 years with complete clinical and laboratory data allowing assignment to the patient or control group were eligible for inclusion.

The study was conducted at the Department of Internal Medicine, Sivas Cumhuriyet University Faculty of Medicine, between January 1, 2020 and December 31, 2024. A total of 184 individuals were included, comprising 92 patients and 92 age- and sex-matched healthy controls.

The study protocol was approved by the Sivas Cumhuriyet University Health Sciences Research Ethics Committee (Approval No: 2025-02/121; Date: February 20, 2025) and was conducted in accordance with the principles of the Declaration of Helsinki. All data were obtained retrospectively from medical record reviews.

In all participants, thyroid function tests (free thyroxine [FT4, ng/dL] and thyroid-stimulating hormone [TSH, mIU/L]), thyroid autoantibodies (anti-TG [IU/mL] and anti-TPO [IU/mL], inflammatory markers (C-reactive protein [CRP, mg/L] and erythrocyte sedimentation rate [ESR, mm/h] and complete blood count parameters, including leukocyte counts [10³/L], neutrophil counts [10³/L], lymphocyte counts [10³/L], monocyte counts [10³/L], platelet counts [10³/L], hemoglobin [g/dL], red cell distribution width [RDW, %] and as a biochemical test albumin (g/dL) were evaluated. Laboratory results were interpreted in accordance with the institutional laboratory’s reference ranges. Serum levels of TSH and FT4 were measured using the Roche Cobas e801 analyzer via electrochemiluminescence immunoassay (ECLIA). The institutional reference ranges were 0.27–4.2 µIU/mL for TSH and 0.93–1.7 ng/dL for FT4.

The following indices and ratios were calculated from hematological and biochemical parameters: NLR, PLR, SII ([platelet × neutrophil]/lymphocyte), RDW-to-lymphocyte ratio (RLR), LMR, CRP-to-albumin ratio (CAR), and the FT4/TSH ratio.

### Statistical analysis

Statistical analyses were performed using the RStudio environment and IBM SPSS Statistics for Windows, version 27.0 (IBM Corp., Armonk, NY, USA). Continuous variables were summarized as the mean ± standard deviation (X̄ ± SD) or the median with interquartile range (IQR), depending on the distribution of the data. In contrast, categorical variables were expressed as counts and percentages. Normality assumptions were assessed, and between-group comparisons were conducted using appropriate parametric (independent-samples t-test, one-way ANOVA) or nonparametric (Mann–Whitney U test, Kruskal–Wallis H test) tests, as applicable. All statistical tests were two-tailed, and a *p* value < 0.05 was considered statistically significant.

The discriminatory performance of selected biomarkers was evaluated using receiver operating characteristic (ROC) curve analysis. Area under the curve (AUC) values with 95% confidence intervals were reported. The statistical significance of AUC values was assessed by testing whether the AUC differed from 0.5 (no discrimination), and only statistically significant results (*p* < 0.05) were reported. Optimal cut-off values were determined using the Youden index, and corresponding sensitivity and specificity values were calculated. To identify independent predictors of patient–control status, a multivariable logistic regression model was constructed. NLR, PLR, SII, RLR, LMR, CAR and the FT4/TSH ratio were included in the model as continuous variables. Model results were reported as regression coefficients (β), odds ratios (OR = exp[β]), and 95% confidence intervals. To improve interpretability, odds ratios were also considered per one standard deviation (SD) increase for continuous predictors. Figures and tables were generated using RStudio’s standard output formats.

## Results

Regarding demographic characteristics, the control group was evenly split between women and men (50% each), whereas the patient group was predominantly women (81.5%), with men accounting for 18.5%. The mean age was 40.14 ± 5.27 years in the control group and 40.94 ± 16.45 years in the patient group. Patients with Hashimoto’s thyroiditis were further classified according to thyroid functional status into overt hypothyroid, subclinical hypothyroid, and euthyroid subgroups for subgroup analyses (*n* = 28, *n* = 37, and *n* = 27, respectively).

As shown in Table 1, the patient group exhibited significant thyroid dysfunction and autoimmunity, with markedly altered TSH, FT4-related ratios, and thyroid autoantibody levels compared with controls (all *p* < 0.001). Inflammatory assessment revealed a significantly higher ESR (*p* < 0.001), whereas CRP and albumin levels were comparable between groups (*p* = 0.081 and *p* = 0.331, respectively). Hematological parameters were broadly similar; however, patients had lower hemoglobin levels (*p* < 0.001), higher monocyte counts (*p* = 0.008), and a lower LMR (*p* < 0.001). Composite inflammatory indices, including NLR, PLR, SII, and CAR, did not differ significantly between groups (all *p* values > 0.05), suggesting a selective rather than generalized inflammatory response.

**Table 1:** Comparison of biochemical values and inflammation markers between the patient and healthy groups

	Control (*n* = 92)	Patient (*n* = 92)	*p*
FT4	1.18 (1.10–1.34)	1.11 (0.86-1.27)	0.001*
TSH	1.79 (1.21–2.61)	6.55 (2.83–16.47)	< 0.001*
Anti-TG	15.60 (13.62–18.35)	272.50 (91.70-453.64)	< 0.001*
Anti-TPO	11.10 (9.00-12.55)	181.00 (94.92-229.75)	< 0.001*
CRP	2.71 (1.11–3.73)	1.85 (0.77-3.55)	0.081
Albumin	4.50 (4.30–4.63)	4.53 (4.39–4.71)	0.331
ESR	6.00 (3.00-8.75)	15.30 (6.00–16.00)	< 0.001*
Leukocyte count	7.08 ± 1,54	7,23 ± 1,94	0.573
Neutrophil count	4.13 (3.09–4.99)	3.90 (3.10–5.05)	0.970
Lymphocyte count	2,32±,52	2,27±,68	0.569
Monocyte count	0.44 (0.35-0.54)	0.49 (0.39-0.63)	0.008*
Hemoglobin	14,55 ± 1,63	13,09 ± 1,91	< 0.001*
RDW	13.60 (13.00-14.10)	13.60 (12.60–14.80)	0.866
Platelet count	282,45 ± 76,71	288,57 ± 67,37	0.699
NLR	1.74 (1.36–2.21)	1.84 (1.34–2.23)	0.480
PLR	118.41 (96.89-149.12)	124. 17 (100.61-163.83)	0.186
SII	468.90 (375.18-608.51)	501.62 (382.36-666.61)	0.271
RLR	5.95 (5.09–6.85)	5.95 (4.91–8.46)	0.424
LMR	5,55 ± 1,81	4,64 ± 1,46	<,001*
CAR	0.56 (0.24-0.81)	0.43 (0.16-0.78)	0.075
FT4/TSH	0.64 (0.46-1.04)	0.18 (0.06-0.39)	< 0.001*

Values are presented as mean ± SD or median (interquartile range), as appropriate. Comparisons between groups were performed using the independent samples *t*-test or Mann–Whitney U test. A *p* value < 0.05 was considered statistically significant (*). FT4, free thyroxine; TSH, thyroid-stimulating hormone; Anti-TG, anti-thyroglobulin antibody; Anti-TPO, anti-thyroid peroxidase antibody; CRP, C-reactive protein; ESR, erythrocyte sedimentation rate; RDW, red cell distribution width; NLR, neutrophil-to-lymphocyte ratio; PLR, platelet-to-lymphocyte ratio; SII, systemic immune-inflammation index; RLR, red cell distribution width-to-lymphocyte ratio; LMR, lymphocyte-to-monocyte ratio; CAR, C-reactive protein-to-albumin ratio

**Fig. 1:**
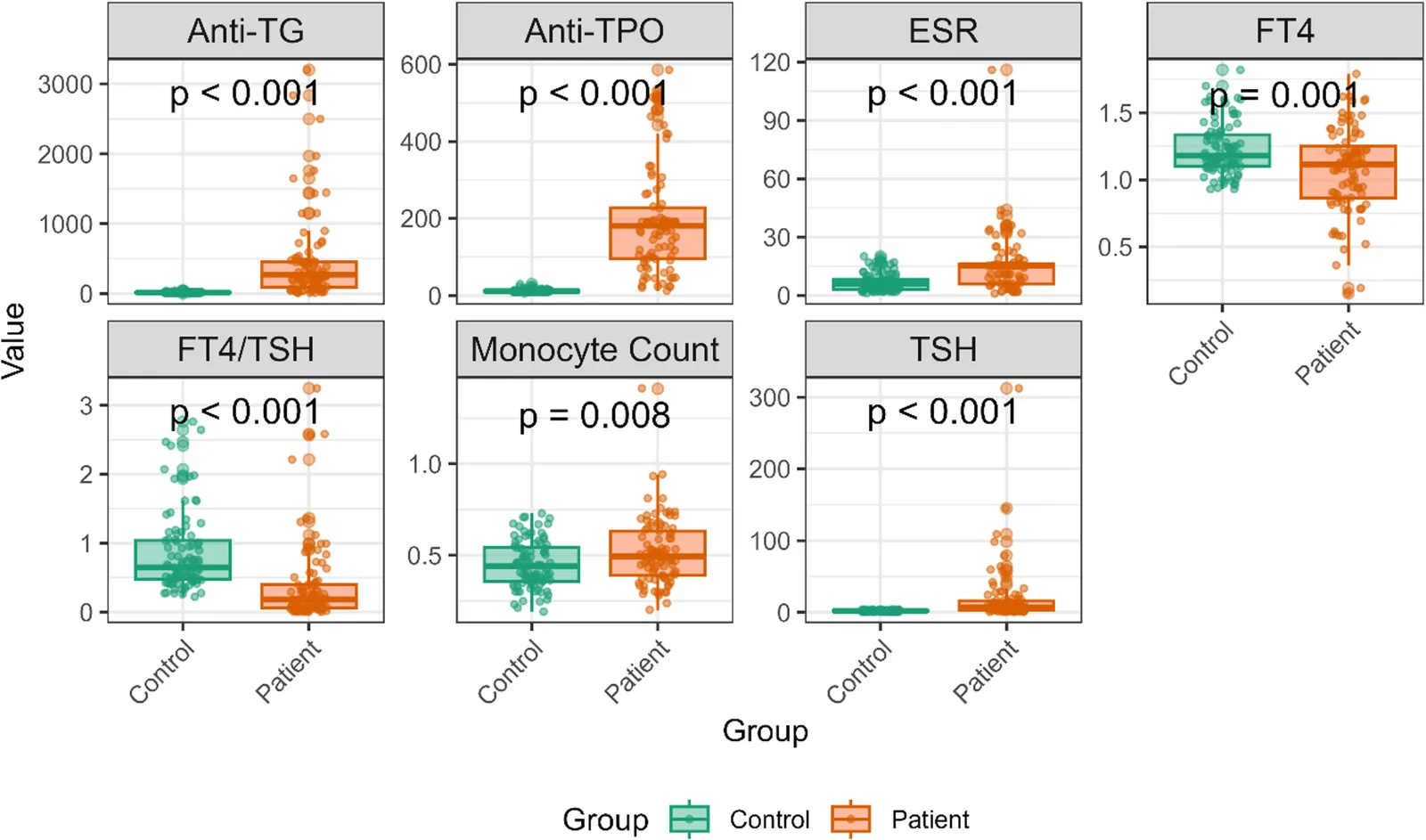
Distribution of thyroid-related and inflammatory parameters in patient and control groups: Box plots with overlaid individual data points illustrate the distribution of Anti-TG, Anti-TPO, T4, TSH, T4/TSH ratio, erythrocyte sedimentation rate (ESR), and monocyte count in patients and controls. Data are presented as median and interquartile range (IQR). The horizontal line within each box represents the median, boxes indicate the IQR, and whiskers denote the minimum and maximum values. Between-group comparisons were performed using appropriate statistical tests, and statistically significant differences are indicated (*p* < 0.05)

**Fig. 2:**
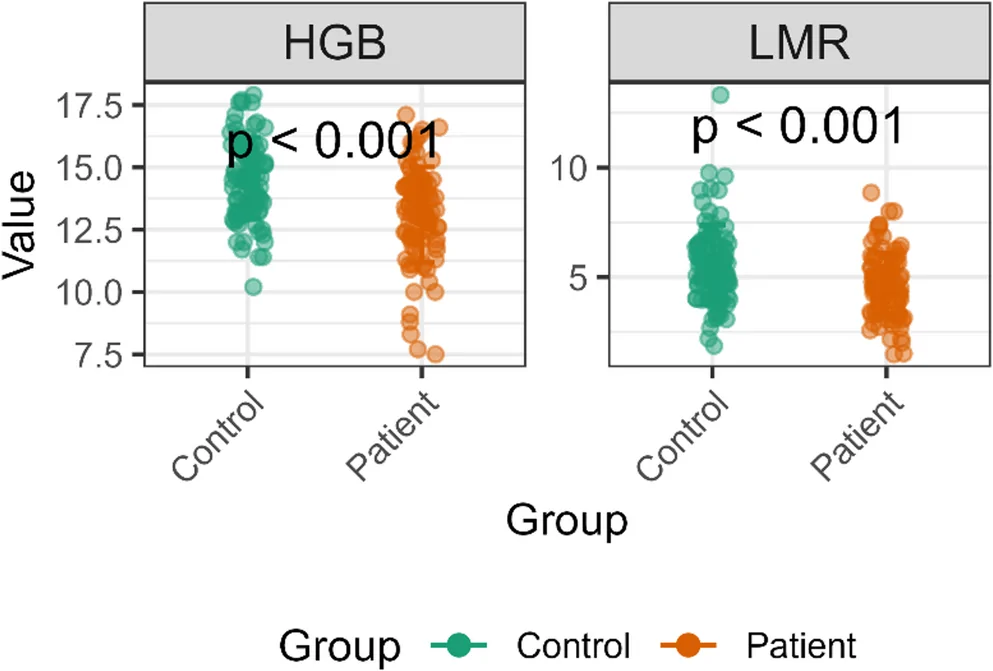
Distribution of hemoglobin and lymphocyte-to-monocyte ratio in patient and control groups: Mean values with standart deviation (X̄ ± SD) are shown for hemoglobin levels and the lymphocyte-to-monocyte ratio (LMR) in patients and controls. Error bars represent standard deviations. Individual data points may be overlaid. Between-group comparisons were performed using appropriate statistical tests, and statistically significant differences are indicated (*p* < 0.05)

Figures 1 and 2 illustrate the distribution of parameters that differed significantly between the patient and control groups. Anti-TG and Anti-TPO levels were markedly higher in patients, with higher medians, wider interquartile ranges, and right-skewed distributions (both *p* < 0.001). Thyroid function parameters demonstrated a reciprocal pattern, with lower FT4 levels (*p* = 0.001) and higher TSH concentrations (*p* < 0.001) in patients, resulting in a significantly reduced FT4/TSH ratio (*p* < 0.001). ESR (*p* < 0.001) and monocyte count (*p* = 0.008) were modestly but significantly elevated in the patient group, accompanied by increased variability.

As shown in Fig. 2, hemoglobin levels and the LMR were significantly lower in patients than in controls (both *p* < 0.001). These parameters showed a downward shift in their distributions, with greater dispersion in the patient group. Overall, autoantibody and thyroid-related parameters showed limited overlap between groups, whereas inflammatory and hematological markers exhibited broader distributions between patients and controls.

In the subgroup analysis, thyroid function parameters differed significantly across overt hypothyroid, subclinical hypothyroid, and euthyroid groups (Table 2). Progressive increases in FT4 levels and corresponding decreases in TSH concentrations were observed from overt hypothyroidism to euthyroidism, resulting in a marked stepwise increase in the FT4/TSH ratio (all *p* values < 0.001). In contrast, thyroid autoantibody levels, inflammatory markers, hematological parameters, and composite inflammatory indices were comparable across the three groups, with no statistically significant differences (*p* > 0.05 for all).

**Table 2:** Comparison of thyroid function, autoantibody levels, inflammatory markers and hematological parameters among overt hypothyroid, subclinical hypothyroid and euthyroid patient subgroups

	Overt hypothyroid (*n* = 28)	Subclinical hypothyroid (*n* = 37)	Euthyroid (*n* = 27)	*p*
FT4	0.78 (0.58-0.85)	1.17 (1.06–1.28)	1.28 (1.13–1.44)	< 0.001*
TSH	21.10 (11.72–55.67)	6.64 (4.88–12.40)	1.65 (1.27–2.57)	< 0.001*
Anti-TG	300.00 (48.00-503.75)	260.00 (92.51–602.30)	304.00 (179.00-453.64)	0.916
Anti-TPO	179.64 (92.10–255.00)	191.28 (111.00-262.50)	156.00 (59.10-191.28)	0.335
CRP	1.52 (0.73-2.67)	2.05 (0.91-4.35)	1.86 (0.66-3.11)	0.592
Albumin	4.53 (4.34–4.77)	4.53 (4.41–4.67)	4.53 (4.42–4.71)	0.742
ESR	15.30 (6.75–15.82)	15.30 (6.00-20.50)	15.30 (6.00-15.30)	0.929
Leukocyte count	7.60 (6.01–9.25)	6.67 (5.82–7.38)	7.18 (5.75–8.47)	0.151
Neutrophil count	4.17 (3.25–5.52)	3.78 (2.69–4.41)	4.30 (3.33–5.12)	0.261
Lymphocyte count	2.43±0.82	2.22±0.58	2.18±0.66	0.356
Monocyte count	0.58 ± 0.22	0.48±0.14	0.50±0.18	0.075
Hemoglobin	13.15 (11.40–14.20)	13.30 (12.55–14.25)	13.2 (12.4–14.3)	0.690
RDW	13.95 (13.20-16.57)	13.30 (12.60-14.55)	13.5 (12.6–14.3)	0.160
Platelet count	277.60 ± 69.48	282.00 ± 60.82	302.14 ± 73.36	0.352
NLR	1.84 (1.38–2.22)	1.73 (1.27–2.16)	1.95 (1.34–2.31)	0.559
PLR	117.43 (83.51-148.47)	121.79 (102.70-154.47)	138.4 (115.06-170.29)	0.115
SII	519.47 (388.18-608.16)	457.67 (374.12-647.57)	560.35 (377.64-738.98)	0.288
RLR	6.09 (4.35–8.46)	5.91 (5.25–7.37)	6 (4.9–8.81)	0.929
LMR	4.45 ± 1.62	4.87 ± 1.61	4.51 ± 1.00	0.463
CAR	0.33 (0.16-0.60)	0.45 (0.20-0.97)	0.42 (0.12-0.76)	0.604
FT4/TSH	0.03 (0.01-0.07)	0.18 (0.09-0.24)	0.72 (0.43-1.11)	< 0.001*

Values are presented as mean ± standard deviation or median (interquartile range), as appropriate. Comparisons among the three groups were performed using one-way ANOVA or the Kruskal–Wallis test. A *p* value < 0.05 was considered statistically significant. FT4, free thyroxine; TSH, thyroid-stimulating hormone; Anti-TG, anti-thyroglobulin antibody; Anti-TPO, anti-thyroid peroxidase antibody; CRP, C-reactive protein; ESR, erythrocyte sedimentation rate; RDW, red cell distribution width; NLR, neutrophil-to-lymphocyte ratio; PLR, platelet-to-lymphocyte ratio; SII, systemic immune-inflammation index; RLR, RDW-to-lymphocyte ratio; LMR, lymphocyte-to-monocyte ratio; CAR, C-reactive protein-to-albumin ratio

In the comparison of clinical, subclinical, and euthyroid subgroups of Hashimoto’s thyroiditis, thyroid hormone–related parameters differed significantly across disease stages. Serum FT4 levels, TSH concentrations, and the FT4/TSH ratio showed statistically significant differences among the three groups (all *p* values < 0.001), indicating that hormonal profiles can clearly distinguish the clinical spectrum of the disease. In contrast, thyroid autoantibody levels and inflammatory markers did not differ significantly between subgroups (all *p* values > 0.05), suggesting a comparable autoimmune and inflammatory background across clinical stages.

**Fig. 3:**
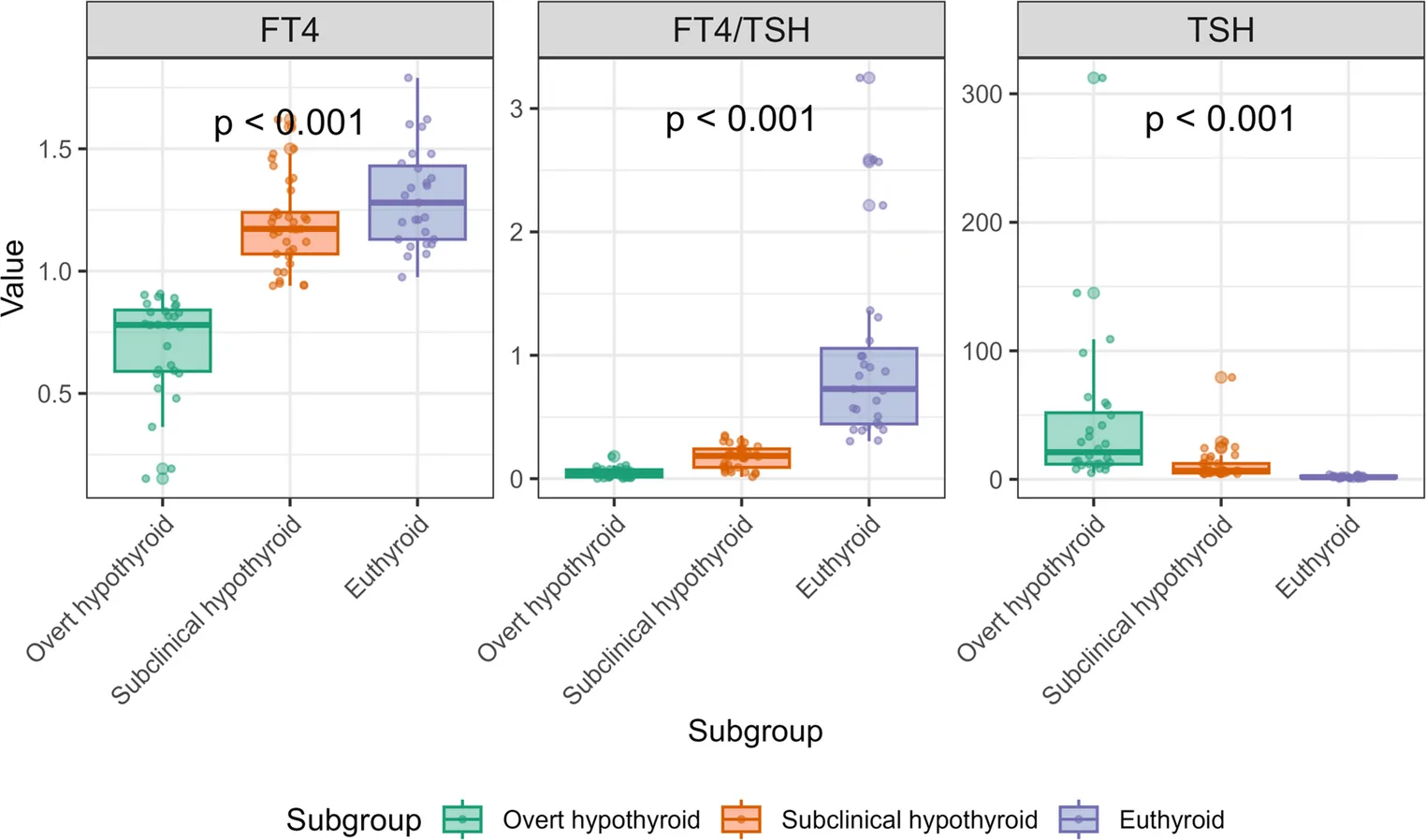
Distribution of T4, TSH, and T4/TSH ratio across overt hypothyroid, subclinical hypothyroid, and euthyroid subgroups of Hashimoto’s thyroiditis: Box plots with overlaid individual data points illustrate the distribution of serum T4, TSH, and the T4/TSH ratio across clinical hypothyroid, subclinical hypothyroid, and euthyroid subgroups of Hashimoto’s thyroiditis. Data are presented as median and interquartile range (IQR). The horizontal line within each box represents the median, boxes indicate the IQR, and whiskers denote the minimum and maximum values. Between-group comparisons were performed using the Kruskal–Wallis test, and statistically significant differences are indicated (*p*<0.05)

To illustrate these differences, box plots of parameters showing significant variation among subgroups are presented in Fig. 3. As disease status progressed from clinical hypothyroidism to the euthyroid state, FT4 levels increased and TSH concentrations decreased. Accordingly, the FT4/TSH ratio was markedly higher in the euthyroid subgroup with minimal overlap with the clinical subgroup. The clinical subgroup showed the widest and right-skewed TSH distribution, indicating variability in disease severity.

**Fig. 4:**
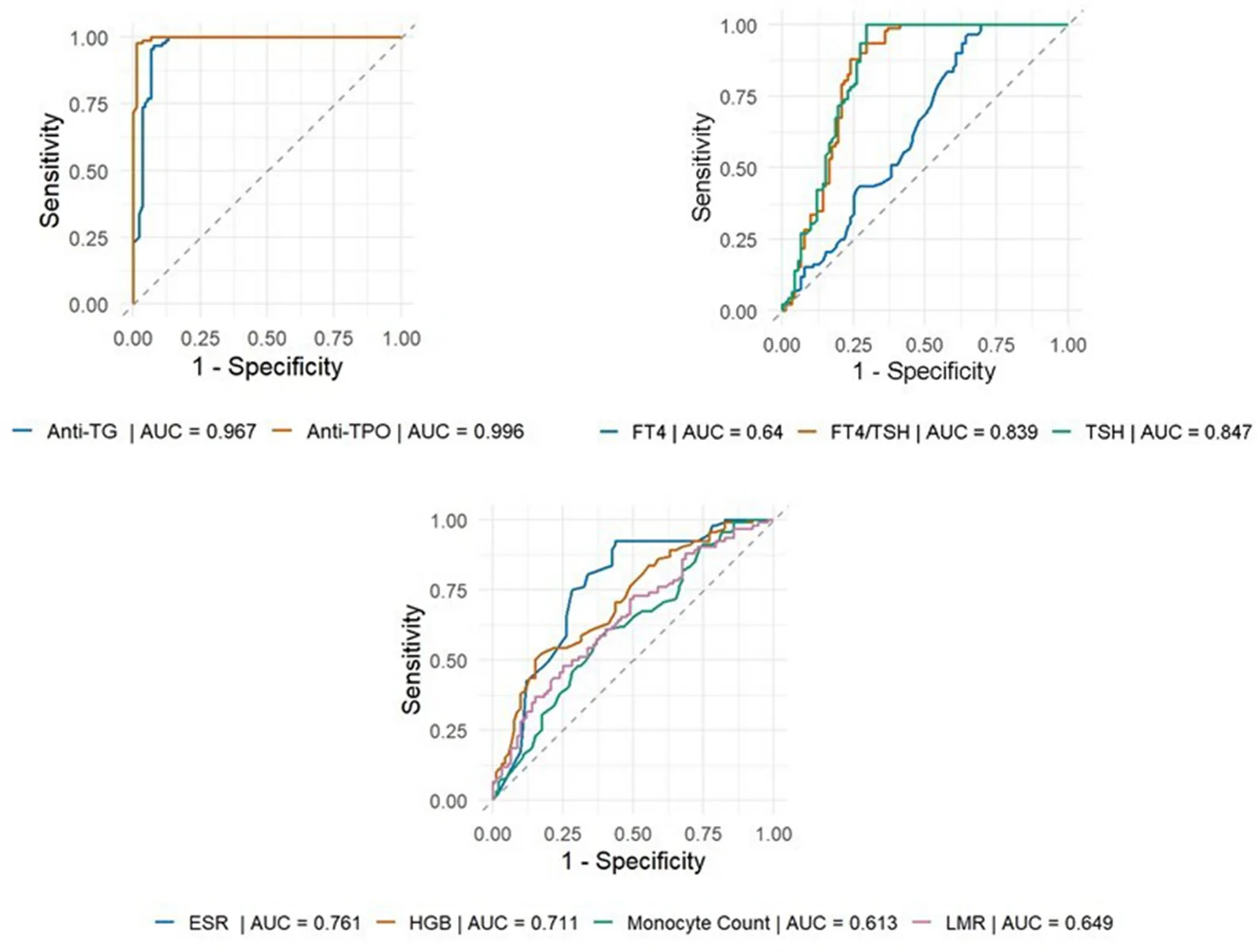
Diagnostic Performance of Autoantibodies, Thyroid Hormones, and Inflammatory Indices in Hashimoto Thyroiditis: Receiver operating characteristic (ROC) curves showing the diagnostic performance of selected biomarkers in differentiating patients with Hashimoto’s thyroiditis from controls. Curves are presented for thyroid autoantibodies, thyroid function parameters, and selected inflammatory and hematological markers. Corresponding area under the curve (AUC) values are indicated, and the dashed diagonal line represents no discriminative ability. *Abbreviations*: TSH, thyroid-stimulating hormone; T4, thyroxine; LMR, lymphocyte-to-monocyte ratio; SII, systemic immune-inflammation index; ROC, receiver operating characteristic

The diagnostic performance of selected biomarkers in distinguishing patients from controls was evaluated using ROC curve analysis. ROC curves and corresponding AUC values are presented in Fig. 4. Thyroid autoantibodies demonstrated high discriminatory ability for patient–control differentiation. Thyroid function parameters showed moderate-to-high diagnostic performance, whereas inflammatory and hematological markers exhibited more limited discriminative capacity. These findings indicate that autoantibodies provide the primary diagnostic contribution, with other parameters offering complementary value.

Table 3 summarizes the diagnostic performance of selected biomarkers for Hashimoto’s thyroiditis as evaluated by ROC curve analysis. Only parameters showing statistically significant discriminatory ability were included in the study. Thyroid autoantibodies demonstrated excellent diagnostic accuracy. Anti-TG and Anti-TPO showed very high AUC values (0.967 and 0.996, respectively), indicating a strong ability to distinguish patients from controls. The optimal cut-off value for Anti-TG was 30.95, yielding a sensitivity of 96.7% and a specificity of 92.4%. For Anti-TPO, the cut-off value of 19.6 provided a sensitivity of 97.8% and a specificity of 98.9%.

Among thyroid function–related parameters, TSH and the FT4/TSH ratio also exhibited good diagnostic performance (AUC = 0.847 and 0.839, respectively), supporting the clinical relevance of conventional thyroid function tests. A TSH cut-off value of 4.21 achieved 100% sensitivity and 70.7% specificity, while a T4/TSH ratio cut-off of 0.318 yielded a sensitivity of 93.5% and a specificity of 70.7%.

In contrast, hematological and inflammatory parameters, including hemoglobin level, monocyte count, and LMR, showed moderate discriminatory capacity. These findings suggest that such markers are more appropriately considered as complementary biomarkers rather than standalone diagnostic tools in Hashimoto’s thyroiditis.

**Table 3:** ROC analysis of biochemical score results predicting Hashimoto’s thyroiditis

	Cutpoint	Sensitivity	Specificity	PPV	NPV	Youden	AUC	95% CI(Lower-Upper)
Anti-TG	30.950	0.967	0.924	0.927	0.966	0.891	0.967*	0.938–0.995
Anti-TPO	19.600	0.978	0.989	0.989	0.978	0.967	0.996*	0.990-1.000
FT4	0.965	0.957	0.359	0.599	0.892	0.315	0.640*	0.559–0.721
TSH	4.210	1.000	0.707	0.773	1.000	0.707	0.847*	0.786–0.908
FT4/TSH	0.318	0.935	0.707	0.761	0.915	0.641	0.839*	0.777–0.901
ESR	15.152	0.924	0.565	0.680	0.881	0.489	0.761*	0.689–0.832
Hemoglobin	14.550	0.522	0.826	0.750	0.633	0.348	0.711*	0.638–0.785
Monocyte count	0.465	0.609	0.587	0.596	0.600	0.196	0.613*	0.532–0.694
LMR	4.572	0.728	0.500	0.593	0.648	0.228	0.649*	0.570–0.728

*p < 0.05

Overall, these findings indicate that Hashimoto’s thyroiditis is a complex disorder that is not limited to thyroid hormone imbalance but also involves prominent autoimmune and inflammatory components. While autoantibodies and hormonal parameters emerge as the most robust diagnostic indicators, hematological and inflammatory indices appear to provide complementary biomarkers that reflect the systemic effects of the disease and contribute to clinical assessment.

**Table 4:** Adjusted odds ratios for patient vs. control status according to inflammation-derived indices and the FT4/TSH ratio

	β (95% CI)	OR (95% CI)	*p*
(Intercept)	3.401 (1.200-5.603)	30.0213 (3.3218-271.3232)	0.002*
NLR	-1.201 (-0.2479-0.077)	0.3009 (0.0838-1.0806)	0.066
PLR	-0.016 (-0.036-0.004)	0.9843 (0.9644-1.0047)	0.131
SII	0.004-(0.000-0.008)	1.0039 (0.9997-1.0081)	0.069
RLR	0.289 (-0.073-0.651)	1.3346 (0.9289-1.9174)	0.118
LMR	-0.394 (-0.656-0.131)	0.6743 (0.5186-0.8766)	0.003*
CAR	-0.066 (-0.383-0.251)	0.9360 (0.6813-1.2861)	0.683
FT4/TSH	-1.727 (-2.521-(-0.932))	0.1779 (0.0804-0.3937)	0.000*

1 $$\begin{aligned}&\mathrm{l}\mathrm{o}\mathrm{g}\mathrm{i}\mathrm{t}\left(\mathrm{p}\right)=3.401-1.201\left(\mathrm{N}\mathrm{L}\mathrm{R}\right)-0.016\left(\mathrm{P}\mathrm{L}\mathrm{R}\right)+0.004\left({\mathrm{S}\mathrm{I}\mathrm{I}}_{1}\right)+0.289\\&\left(\mathrm{R}\mathrm{L}\mathrm{R}\right)-0.394\left(\mathrm{L}\mathrm{M}\mathrm{R}\right)-0.066\left(\mathrm{C}\mathrm{A}\mathrm{R}\right)-1.727\left(\mathrm{F}\mathrm{T}4/\mathrm{T}\mathrm{S}\mathrm{H}\right)\end{aligned}$$

The multivariable logistic regression model describing the probability of patient status is shown in Eq. 1, with regression coefficients and corresponding odds ratios summarized in Table 4. Among the included parameters, the LMR and the FT4/TSH ratio were independently and significantly associated with patient status. LMR showed a negative association (β = −0.394, 95% CI: −0.656 to − 0.131), corresponding to an OR of 0.674 (95% CI: 0.519–0.877; *p* = 0.003). Each one-unit increase in LMR was associated with an approximate 33% reduction in the odds of being in the patient group. Similarly, the FT4/TSH ratio showed a strong inverse association with patient status (β = −1.727; OR = 0.178, 95% CI: 0.080–0.394; *p* < 0.001), indicating an approximately 82% decrease in the odds of patient status per unit increase.

NLR and SII showed borderline associations with patient status (OR = 0.301, *p* = 0.066; OR = 1.004, *p* = 0.069, respectively), with confidence intervals that included unity. PLR, RLR, and CAR were not independently associated with patient status, as indicated by non-significant *p* values and confidence intervals that crossed 1.

## Discussion

This study demonstrates that Hashimoto’s thyroiditis is associated with pronounced alterations in thyroid-related parameters and selective changes in inflammation-derived hematological indices. Patients showed markedly higher thyroid autoantibody levels and distinct thyroid hormone profiles compared with healthy controls, confirming the substantial diagnostic value of these markers. In addition, ESR, monocyte count, hemoglobin level and the LMR differed significantly between groups, indicating disease-related immune and hematological involvement. Subgroup analyses further revealed that thyroid hormone–related parameters effectively distinguished clinical disease stages, whereas autoantibody levels and most inflammatory indices remained comparable across thyroid functional states.

As expected, thyroid autoantibodies and conventional thyroid function tests reflected the well-established biochemical characteristics of the disease. A notable observation of the present study is the strong diagnostic performance of the FT4/TSH ratio, which integrates reciprocal alterations in thyroid hormones into a single composite parameter. While TSH and FT4 are routinely interpreted independently, ratio-based approaches may better capture subtle shifts in hypothalamic-pituitary–thyroid (HPT) axis dynamics. In the existing literature, this ratio has been investigated predominantly in hyperthyroidism [8, 9], with limited data available for hypothyroid states. However, emerging evidence suggests that dysregulation of thyroid hormone responsiveness may be present even in euthyroid patients [10].

The clinical added value of the FT4/TSH ratio, compared to the separate interpretation of TSH and FT4, lies in its ability to reflect the integrated sensitivity and equilibrium of the HPT axis. While TSH and FT4 are standard diagnostic markers, they represent isolated components of a feedback loop. By evaluating them as a ratio, we can better account for the dynamic compensation between pituitary stimulation and thyroid output. In our cohort, this ratio provided a more nuanced differentiation of thyroid functional states, particularly in cases where individual parameters might remain within borderline reference ranges but the overall HPT set-point is already displaced. Thus, the FT4/TSH ratio serves as a sensitive physiological indicator of thyroid functional reserve and homeostatic disruption in autoimmune thyroiditis.

Beyond hormonal parameters, inflammation-derived indices demonstrated a selective pattern. Patients showed higher ESR and monocyte counts alongside lower hemoglobin and LMR, whereas composite indices such as NLR, PLR, and SII did not differ significantly. This suggests that immune activation in Hashimoto’s thyroiditis manifests as a chronic, low-grade process rather than a uniform systemic response. Similar observations in other autoimmune endocrine disorders indicate that single-cell–based parameters may be more sensitive indicators of immune dysregulation than global composite scores [6, 11]. Current immunological models emphasize persistent lymphocytic infiltration and tissue-level redistribution over overt systemic inflammation [12], which may explain the limited performance of global scores. In line with this, Duman et al. reported significant alterations in CAR but inconsistent results for NLR and PLR [13].

In subgroup analyses, when patients with Hashimoto’s thyroiditis were classified as overt hypothyroid, subclinical hypothyroid, and euthyroid, thyroid hormone–related parameters clearly distinguished the clinical stages of the disease. Serum FT4 levels showed a gradual increase from overt hypothyroidism to the euthyroid state, while TSH concentrations decreased accordingly. This reciprocal pattern indicates that thyroid functional impairment remains the primary determinant of the clinical spectrum of Hashimoto’s thyroiditis. In parallel, the progressive increase in the FT4/TSH ratio from the clinical to the euthyroid stage suggests that this ratio may provide an integrated reflection of thyroid functional status.

In contrast, thyroid autoantibody levels (anti-TPO and anti-TG) did not differ significantly among clinical, subclinical, and euthyroid subgroups. This finding supports the concept that thyroid autoimmunity may persist independently of clinical disease stage and that the presence of autoantibodies does not necessarily parallel the degree of hormonal dysfunction. Previous studies have suggested that thyroid autoantibody levels do not consistently correlate with clinical disease severity and may instead reflect the presence and extent of underlying autoimmunity rather than thyroid functional impairment [14, 15].

Likewise, inflammation-derived hematological indices were broadly comparable across subgroups, suggesting that the inflammatory response in Hashimoto’s thyroiditis may be more closely related to the underlying chronic autoimmune process than to clinical disease stage. It has been emphasized that inflammation in Hashimoto’s thyroiditis is predominantly localized to thyroid tissue. In contrast, systemic inflammatory activity often remains limited [16]. Supporting this concept, recent studies have shown that systemic inflammatory markers do not differ significantly between euthyroid and hypothyroid subgroups of Hashimoto’s thyroiditis, despite evidence of ongoing immune activation at the tissue level [17, 18]. Proteomic profiling studies have further demonstrated inflammatory alterations in patients with Hashimoto’s thyroiditis, without clear stratification by thyroid functional status [19].

Taken together, these findings indicate that thyroid hormone parameters are the principal determinants of clinical staging in Hashimoto’s thyroiditis. In contrast, autoimmune and inflammatory markers appear to reflect a more stable immunological background that is largely independent of thyroid functional status.

When the results of the ROC analysis and multivariable logistic regression are considered together, a two-layered approach appears to be meaningful in clinical practice. Thyroid-related parameters accurately identify disease, whereas selected complete blood count–derived indices provide additional information regarding immune imbalance. In the adjusted logistic regression model, both the LMR and the FT4/TSH ratio were independently associated with patient status, indicating that these parameters contribute complementary diagnostic value beyond other inflammation-derived indices included in the model (Eq. 1).

These findings are consistent with the existing literature showing variable performance of composite indices, such as the NLR and PLR, in Hashimoto’s thyroiditis. While some studies have reported significant differences in these indices between patients and healthy controls, others have demonstrated limited or inconsistent results depending on clinical context and population characteristics [20, 21].

This variability suggests that prioritizing the most informative indices within multivariable models may offer a more robust and generalizable strategy in real-world clinical settings.

Moreover, it is well recognized that inflammation-derived hematological markers may behave differently across patient groups. In adult populations with Hashimoto’s thyroiditis, changes in NLR and PLR have been reported inconsistently, whereas studies in pediatric cohorts have shown more pronounced alterations in selected indices. This heterogeneity supports the notion that systemic inflammatory signatures in autoimmune thyroiditis are heterogeneous and may vary with disease phenotype and host characteristics [22].

Within this framework, the emergence of the FT4/TSH ratio and LMR as independent predictors suggests that integrating hormonal imbalance with alterations in immune cell distribution may provide a more clinically meaningful assessment of Hashimoto’s thyroiditis than reliance on either domain alone.

The findings of this study indicate that ratios derived from routinely performed laboratory tests may provide meaningful clinical insight in the evaluation of Hashimoto’s thyroiditis. Rather than relying on novel or costly biomarkers, our results emphasize the diagnostic and interpretive value of simple combinations of standard parameters already used in daily practice. In this context, the FT4/TSH ratio and the LMR emerged as complementary markers reflecting both the hormonal and immune aspects of the disease.

One of the distinguishing features of this study is the observation that these ratios retain independent clinical relevance even when multiple inflammation-derived indices are evaluated simultaneously. As easily calculated and cost-neutral measures, these parameters offer practical advantages, particularly in outpatient settings where Hashimoto’s thyroiditis is commonly managed.

In addition, our findings suggest that not all inflammatory indices contribute equally to clinical decision-making. The selective use of LMR and the FT4/TSH ratio may enable more precise, more individualized assessment, supporting their role as complementary tools alongside established diagnostic measures.

## Limitations

This study has several limitations. First, due to its retrospective and observational design, the findings should be interpreted at an associative level, and conclusions regarding temporal relationships or underlying mechanisms cannot be drawn. Second, since anti-TPO/anti-TG positivity contributed to the diagnostic classification in this retrospective cohort, their near-perfect AUCs may partly reflect incorporation bias, and therefore should be interpreted with caution. Third, the single-center design may limit the generalizability of the results to other populations. Nevertheless, these findings provide insight into the potential contribution of routine laboratory parameters in the evaluation of Hashimoto’s thyroiditis and offer a basis for future prospective studies.

## Conclusion

This study demonstrates that focusing on selected ratios derived from routine laboratory tests, rather than on isolated parameters, can provide meaningful clinical insight into Hashimoto’s thyroiditis. While thyroid autoantibodies and conventional thyroid function tests remain the cornerstone of diagnosis, the FT4/TSH ratio and the LMR have emerged as strong complementary markers that reflect the interplay between hormonal imbalance and immune response.

The selective prominence of these ratios among multiple inflammation-related indices suggests that a more targeted and biologically relevant approach to laboratory evaluation is feasible in Hashimoto’s thyroiditis. By relying on accessible, cost-neutral, and routinely obtained data, this strategy offers a practical framework that may enhance patient assessment in everyday clinical practice.

## Data Availability

The datasets used and/or analyzed during the current study are available from the corresponding author upon reasonable request.
